# Spatially Resolved Molecular Analysis of Host Response to Medical Device Implantation Using the 3D OrbiSIMS Highlights a Critical Role for Lipids

**DOI:** 10.1002/advs.202306000

**Published:** 2024-02-14

**Authors:** Waraporn Suvannapruk, Leanne E Fisher, Jeni C Luckett, Max K Edney, Anna M Kotowska, Dong‐Hyun Kim, David J Scurr, Amir M Ghaemmaghami, Morgan R Alexander

**Affiliations:** ^1^ Advanced Materials and Healthcare Technologies Division School of Pharmacy University of Nottingham University Park Nottingham Nottingham NG7 2RD UK; ^2^ School of Life Sciences Faculty of Medicine and Health Science University of Nottingham University Park Nottingham Nottingham NG7 2RD UK; ^3^ Department of Chemical and Environmental Engineering Faculty of Engineering University of Nottingham University Park Nottingham Nottingham NG7 2RD UK; ^4^ Immunology & Immuno‐bioengineering Group School of Life Sciences Faculty of Medicine and Health Sciences University of Nottingham University Park Nottingham Nottingham NG7 2RD UK; ^5^ Present address: National Metal and Materials Technology Center (MTEC) 114 Thailand Science Park, Phahonyothin Road, Khlong Nueng, Khlong Luang Pathum Thani 12120 Thailand

**Keywords:** 3D OrbiSIMS, immune‐instructive polymers and metabolomics, Macrophage

## Abstract

A key goal for implanted medical devices is that they do not elicit a detrimental immune response. Macrophages play critical roles in the modulation of the host immune response and are the cells responsible for persistent inflammatory reactions to implanted biomaterials. Two novel immune‐instructive polymers that stimulate pro‐ or anti‐inflammatory responses from macrophages in vitro are investigated. These also modulate in vivo foreign body responses (FBR) when implanted subcutaneously in mice. Immunofluorescent staining of tissue abutting the polymer reveals responses consistent with pro‐ or anti‐inflammatory responses previously described for these polymers. Three Dimensional OrbiTrap Secondary Ion Mass Spectrometry (3D OrbiSIMS) analysis to spatially characterize the metabolites in the tissue surrounding the implant, providing molecular histology insight into the metabolite response in the host is applied. For the pro‐inflammatory polymer, monoacylglycerols (MG) and diacylglycerols (DG) are observed at increased intensity, while for the anti‐inflammatory coating, the number of phospholipid species detected decreased, and pyridine and pyrimidine levels are elevated. Small molecule signatures from single‐cell studies of M2 macrophages in vitro correlate with the in vivo observations, suggesting potential for prediction. Metabolite characterization by the 3D OrbiSIMS is shown to provide insight into the mechanism of bio‐instructive materials as medical devices and to inform on the FBR to biomaterials.

## Graphical abstract

Designing a novel noneluting bio‐instructive material to stimulate positive interactions with the immune system by inducing a favorable macrophage response following implantation will be advantageous. The 3D OrbiSIMS technique to conduct molecular histology of mouse tissue abutting implanted bio‐instructive polymers is employed. This ability to obtain untargeted metabolite information from human body reaction to implanted devices can support immune‐instructive biomaterial development for medical devices.

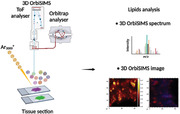

## 1 Introduction

Medical devices are ubiquitous in modern medicine, from coronary stents, catheters, and hip/knee replacements, to everyday contact lenses. Patients can suffer adverse immune reactions to implanted devices, leading to chronic inflammation, pain, and on occasion, implant failure.^[^ ^1^ ^]^ The foreign body response (FBR) and chronic inflammation in the implant microenvironment can be detrimental to the function of implanted materials/tissues and increase patient mortality and morbidity.^[^ ^2^ ^]^ Macrophages play a critical role in orchestrating the FBR to implanted materials,^[^ ^3^ ^]^ and can perpetuate chronic inflammation or enhance tissue healing depending on the phenotype they adopt in response to different biomaterials.^[^ ^4^ ^]^ Therefore, control of inflammatory responses by modulating macrophage phenotype may improve implant integration. This has led to significant interest in designing novel non‐eluting bio‐instructive materials that interact positively with the immune system to induce a favorable macrophage response to medical devices.^[^ ^5^ ^]^

Macrophages are versatile cells that adapt to various signals in their surroundings and develop different functions, exemplified by M1 (pro‐inflammatory) and M2 (anti‐inflammatory) macrophages at either end of a functional spectrum. At the beginning of tissue damage, macrophages assume a pro‐inflammatory state and produce pro‐inflammatory cytokines such as Tumour Necrosis Factor alpha (TNF‐α), Interleukin‐1 beta (IL‐1β) that promote phagocytosis. The pro‐inflammatory macrophages undergo a progressive transition toward anti‐inflammatory morphologies, which are linked to the healing process and the reduction of inflammatory factors. Continued stimulation of M2‐like macrophages can result in fibrosis, as observed in the context of chronic wound healing.^[^ ^5d^ ^]^ Macrophage polarization status and FBR have been the subject of extensive research in recent years. While the immune‐instructive effects of topographical cues are broadly attributed to changes in macrophage mechano‐sensing machinery.^[^ ^6^, ^7^ ^]^

A few studies have investigated the molecular basis of how material chemistry modulates macrophage polarisation status. For example, Doloff et al. investigated the impact of implanted biomaterials on innate and adaptive immune systems in rodents and non‐human primates. Colony‐stimulating factor‐1 receptor (CSF1R) is a central component of the foreign body response to biomaterials, which is significantly increased following implantation. Like macrophage depletion, its inhibition reduces fibrosis but preserves wound healing, reactive oxygen species generation, and phagocytosis.^[^ ^8^ ^]^ More recently Scherib et al., have shown differential deposition of fatty acids or phospholipids on implant surfaces alters FBR by affecting immune cell reactivity to materials and macrophage polarization toward pro or anti‐fibrotic cells. Moreover, in mouse macrophages, phospholipid deposition upregulates anti‐inflammatory genes whereas fatty acid deposition upregulates pro‐inflammatory genes.^[^ ^9^ ^]^ These findings offer more understanding of how different surface chemistries could modulate macrophage phenotype.

In most cases, implants are perceived as foreign bodies by immune cells and can result in chronic and persistent inflammation. Implants can therefore have a profound effect on the host immune response. Polymers have previously been highlighted in many experimental studies to be able to instruct different cell types, influencing attachment density and phenotype.^[^ ^10^ ^]^ New materials that instruct immune responses to promote healing are needed. Screening of polymer libraries has identified cell‐instructive polymers, these can be used as coatings on existing medical devices to mitigate FBR, and enhance device integration and wound healing. For example, Rostam et al. used a high throughput microarray screening method to identify immune‐instructive acrylates and methacrylates able to promote macrophage polarisation toward pro‐inflammatory or anti‐inflammatory phenotypes in vitro. A selection of these polymers was shown to mitigate FBR in subcutaneous implants when used as a thin coating on medical‐grade silicone segments,^[^ ^11^ ^]^ the same method we employ here.

The traditional approach to characterize macrophage phenotype during the FBR relies on immunohistochemistry for markers that are typically associated with pro‐inflammatory and anti‐inflammatory macrophages, such as nitric oxide synthase (iNOS) and the anti‐inflammatory arginise‐1 (Arg‐1) respectively.^[^ ^11^, ^12^ ^]^ One limitation of this approach is the co‐expression of both iNOS and Arg‐1 markers on many macrophages which makes it difficult to determine their functional phenotype accurately, therefore a range of cell surface markers have also been identified by immunohistochemistry to profile macrophages.^[^ ^13^ ^]^ As an alternative to immunohistochemistry, here we investigate an approach using the metabolomic signature of cells and tissues to identify changes within the small molecule population at the host/material interface and provide insight into the related molecular changes within the tissue.

Metabolomics is defined as the comprehensive analysis of metabolites in a biological sample and is a powerful technique that has the potential to improve diagnosis and treatment.^[^ ^14^ ^]^ Metabolomic information can provide an in‐depth understanding of complex molecular interactions within biological systems and provide information that relates to cell phenotype.^[^ ^15^ ^]^ Technologies based on metabolomic markers have been established to study disease, here we explore its power for assessing the influence of bio‐instructive implants.

Studies of metabolites in tissues have used a variety of instrumental and data processing techniques based on targeted and/or non‐targeted techniques such as liquid chromatography‐mass spectrometry (LC‐MS), liquid extraction surface analysis‐MS (LESA‐MS), MALDI‐imaging MS and secondary ion MS (SIMS). LC‐MS is the most commonly used analytical method for detecting metabolites.^[^ ^16^ ^]^ Typically, LC‐MS‐based metabolite analyses proceed with the extraction of metabolites from tissue samples. It requires a significant amount of tissue, for example as presented by Woodward et al., using metabolomics to classify brain tumor tissue required at least 10 mg to obtain a sufficient signal.^[^ ^17^ ^]^ The sample is often prepared using solvent extraction of a tissue surface sample to isolate and inject to separate and then ionize analytes of interest.^[^ ^18^ ^]^

A direct analysis alternative is LESA‐MS, used by Meurs et al., to identify metabolites in pediatric ependymoma tumor tissue.^[^ ^19^ ^]^ LESA‐MS has the advantages of sensitivity and high‐resolution mass analyzers, but the limitations of these approaches are their low spatial resolution (500 µm–1 mm).^[^ ^20^ ^]^ Time‐of‐flight secondary ion MS (ToF‐SIMS) has also been used to identify small molecules in biological samples.^[^ ^21^ ^]^ For application in animal studies, Palmquist et al., performed chemical and structural analysis of the bone‐implant interface using ToF‐SIMS.^[^ ^22^ ^]^ However, ToF‐SIMS has been limited by its relatively low mass resolving power and thus has not been applied to metabolite identification due to associated limitations in assignment certainty.^[^ ^23^ ^]^ In the pursuit of greater assignment specificity of ions, a hybrid SIMS instrument with a high‐resolution Orbitrap mass analyzer has been developed, termed 3D OrbiSIMS.^[^ ^24^ ^]^

The Three Dimensional Orbitrap Secondary Ion Mass Spectrometry (3D OrbiSIMS) uses secondary ions that are liberated from a sample by bombardment using a primary ion source. A high potential accelerates the primary ions to impact the surface induce a fragmentation cascade and generate secondary ions.^[^ ^25^ ^]^ Neutral species, as well as secondary ions (+/‐), and electrons, are desorbed from the first few monolayers of the sample. The 3D OrbiSIMS instrument is equipped with both Time of Flight (ToF) and Orbitrap analyzers, which provide high‐performance mass spectrometry and speed advantages respectively. The general concept of an Orbitrap involves capturing secondary ions in a C‐trap and then injecting them orthogonally as a packet into the analyzer, which consists of an outer wall and interior spindle. Voltages are applied between them, causing injected ions to oscillate radially in an electric field. Each ion with a particular m/z ratio will oscillate with both a radial vector and an axial vector (z‐direction); the latter is detected as fluctuations in the continuous current on the potential plates.

3D OrbiSIMS is a direct surface analysis technique that enables the identification of biomolecules in complex samples using intact molecular ions, generated by an argon gas cluster primary ion beam with 2 µm spatial resolution, high mass resolving power (>240 000 for a peak of m/z 200), and excellent mass accuracy (<2 ppm).^[^ ^24^ ^]^ This capability has been demonstrated on a variety of tissue and cell samples, assigning lipids, proteins, amino acids, peptide fragments of proteins, and a selection of other small molecules.^[^ ^26^ ^]^ Furthermore, studies of metabolomics that use 3D OrbiSIMS do not need to use protocols necessary with other techniques including, chemically fixed cells, liquid extraction procedures, antibody‐based cell markers, or staining. Recently, 3D OrbiSIMS imaging has been used to observe the metabolite characteristics in brain tumor tissue samples and to identify clinically relevant molecular metabolism‐driven subgroup‐specific phenotypes, using a sample preparation approach similar to previous studies.^[^ ^19^, ^24^ ^]^

**Figure 1:**
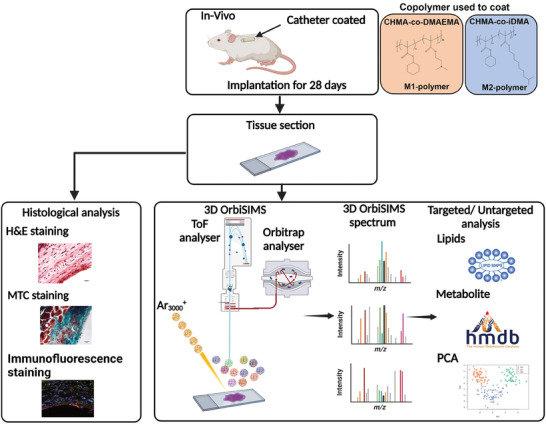
Schematic of the in vivo study experimental procedure. Catheters coated with copolymers were implanted subcutaneously into a mouse model of FBR for 28 days. Following implantation, fresh tissue samples were cut and mounted onto glass slides. For histological analysis, tissue sections were stained with H&E to assess tissue structure, MTC to analyze collagen thickness as an indication of fibrosis, and IHC stains to characterize the macrophage phenotype at the catheter‐tissue interface. For 3D OrbiSIMS, tissue slides were washed with ammonia formate, plunged frozen in liquid nitrogen, and then freeze‐dried. The GCIB was rastered across the tissue section with the Orbitrap analyzer collecting the high‐resolution mass spectrum, followed by multivariate analysis to undertake unbiased sample comparison, complemented with targeted analysis.

Here, we applied 3D OrbiSIMS analysis to investigate whether there was a molecular signature for different pro‐ or anti‐inflammatory macrophages at the interface of tissue‐implanted materials illustrated in **Figure** **1**. We retrieved implants (silicone catheter segments) with or without immune‐instructive polymer coatings described previously^[^ ^11^ ^]^ 28 days after implantation in a subcutaneous murine model using freeze drying prior to analysis. Characteristic metabolites from the histological sections adjacent to the implanted foreign body site were identified using a combination of targeted database approaches to identifying lipids, and a data‐driven multivariate approach to highlight amino acids and other small molecules that we interpreted by comparison with literature on macrophage metabolomics.

## 2 Results and Discussion

### 2.1 Pro‐ and Anti‐Inflammatory Macrophage Instructive Polymers Influence Immune Cell Infiltration and Collagen Deposition in Vivo

Polymers eliciting in vitro polarization of monocytes to M1 pro‐inflammatory macrophage phenotype or to M2 inducing anti‐inflammatory macrophage phenotype were synthesized by thermal free radical polymerization and coated onto segments of clinical‐grade silicone catheter via dipping. The polymers used had previously been identified to polarize macrophages in vitro and modulate FBR in vivo: M1 inducing: poly(cyclohexyl methacrylate‐co‐dimethylamino‐ethyl methacrylate), abbreviated to pCHMA‐DMAEMA induced a pro‐inflammatory macrophage phenotype, and M2 inducing: poly(cyclohexyl methacrylate‐co‐isodecyl methacrylate), abbreviated to pCHMA‐iDMA induced an anti‐inflammatory macrophage phenotype.^[^ ^11^ ^]^ The in vivo responses to these novel polymer coatings were compared to each other and the widely employed biomedical polymer, silicone rubber, by subcutaneous implantation into a mouse model of FBR for 28 days. Upon recovery of the implants and surrounding skin, tissues were snap‐frozen in liquid nitrogen sectioned, and freeze‐dried for 3D OrbiSIMS analysis.

Adjacent sections were stained with haematoxylin and eosin (H&E) and Masson's trichrome (MTC) and immunohistochemically (MIC) labeled with iNOS and Arg‐1 markers before optical microscopy to characterize the inflammatory response, collagen deposition, and phenotype marker respectively.^[^ ^11^ ^]^ The staining and labeling revealed that the total number of macrophages was invariant between the different M1‐polymer and M2‐polymer implants, but higher on the polydimethylsiloxane (PDMS as seen in Figure S1A, Supporting Information, but the anti‐inflammatory coating did result in a far higher M2/M1 ratio of macrophages as 5.6 ± 2.61 in the tissue near the catheter and the pro‐inflammatory coating resulted in a far lower ratio, with the uncoated PDMS implants in between (Figure S1D, Supporting Information).

A lower number of neutrophils were recruited to the M2‐polymer (Figure S1B, Supporting Information), although this was not statistically significant. A lower number on the M2‐polymer would be consistent with phagocytosis‐induced cell death at the earlier stage of the inflammatory response.

The thickness of collagen deposition at the surface of the anti‐inflammatory polymer was significantly greater than the pro‐inflammatory polymer and the uncoated catheters (Figure S1C, Supporting Information). These results are consistent with previous in vivo studies of these polymers undertaken in the same model.^[^ ^11^ ^]^

### 2.2 Characterisation of Metabolite Changes in Tissue Local to Implants Using 3D OrbiSIMS

**Figure 2:**
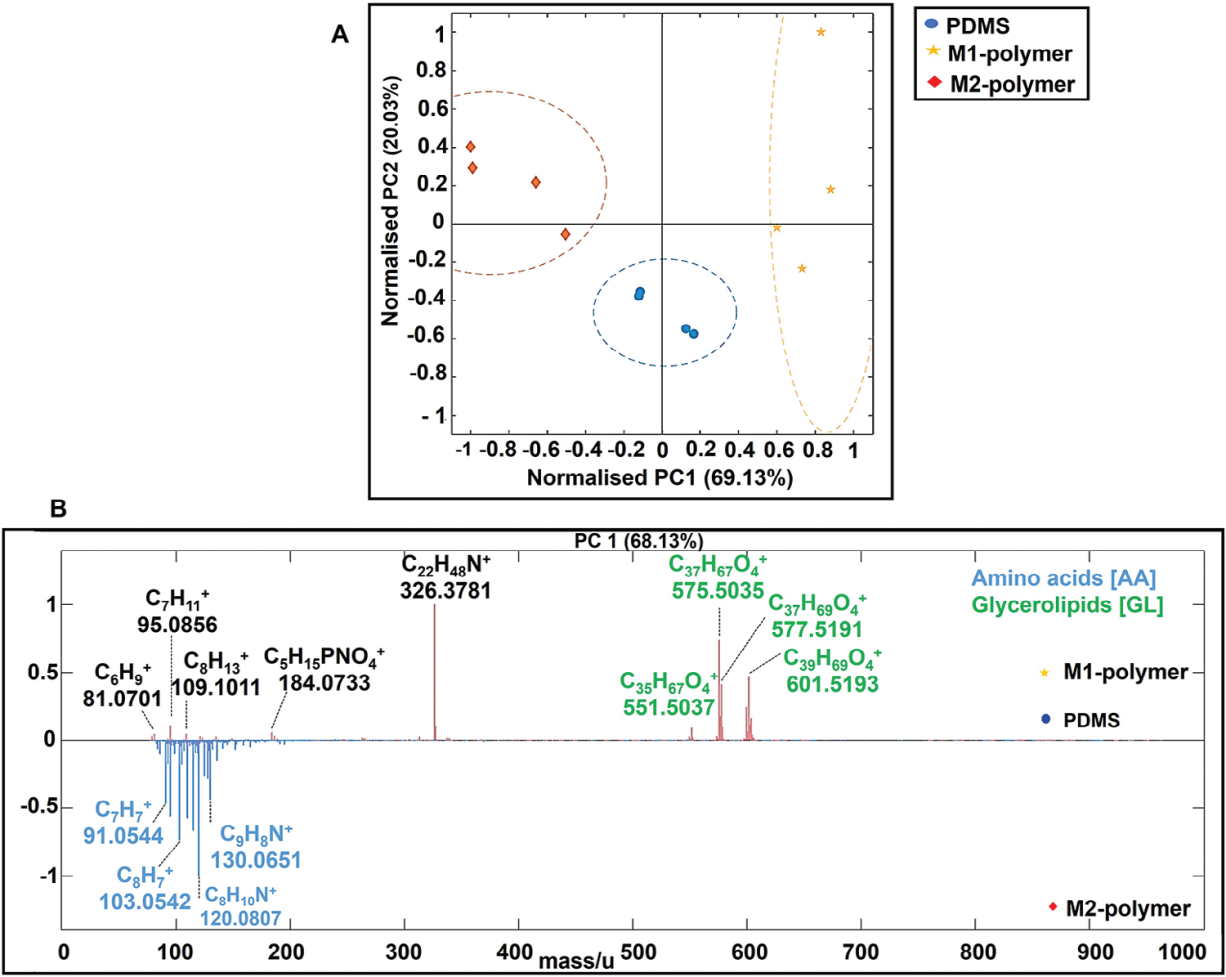
Principal component analysis (scores and loadings) for different tissue samples. A) Principal component scores plot of PC1 and PC2 for the 3D OrbiSIMS spectra of PDMS, M1 polymer, and M2 polymer tissue section samples on positive polarity. B) Principal component analysis of three different tissue samples, loadings plots of the first (PC1) and (PC2) principal components and peak were assigned to glycerolipids (green) and amino acids (blue). The peak at m/z 326.3781 (didecyldimethyl ammonium), is a commercial surface disinfectant unintentionally introduced to the tissue samples somewhere in the sample handling process.

We analyzed the tissue areas surrounding the implant using 3D OrbiSIMS in both positive and negative secondary ion modes to provide molecular histology to delve deeper into the molecular changes coincident with the immunohistochemistry findings. Principal component analysis (PCA) was initially applied as an untargeted data analysis approach to distinguish chemical differences in the complex 3D OrbiSIMS spectra from the local host‐implant interface. The scores plot of the first and second principal components is presented in **Figure** **2**A, highlighting that there are chemical differences between the tissue proximal to the M2‐ and M1‐inducing polymers and the uncoated silicone catheter. The negative score on PC1 was associated with amino acids from the anti‐inflammatory polymer highlighting a series of lower mass peaks, including m/z 91.0545 *m/z* 130.0652 (tryptophan), and m/z 103.0543 m/z 120.0808 (phenylalanine), suggesting higher protein levels in these samples consistent with the greater collagen thickness formed in response to the M2‐inducing polymer implants (Figure S1C, Supporting Information).

Glycerolipids were found to be associated preferentially with the pro‐inflammatory M1‐inducing polymer implants from the *loadings* plot shown in Figure 2B. The positively loaded ions are assigned to glycerol lipids including monoacylglycerols (MG) and diacylglycerols (DG) at m/z 551.5035 (MG 32:2), m/z 575.5035 (MG 34:4), m/z 557.5191(MG 34:3) and m/z 601.5193 (DG O‐36:5).

**Figure 3:**
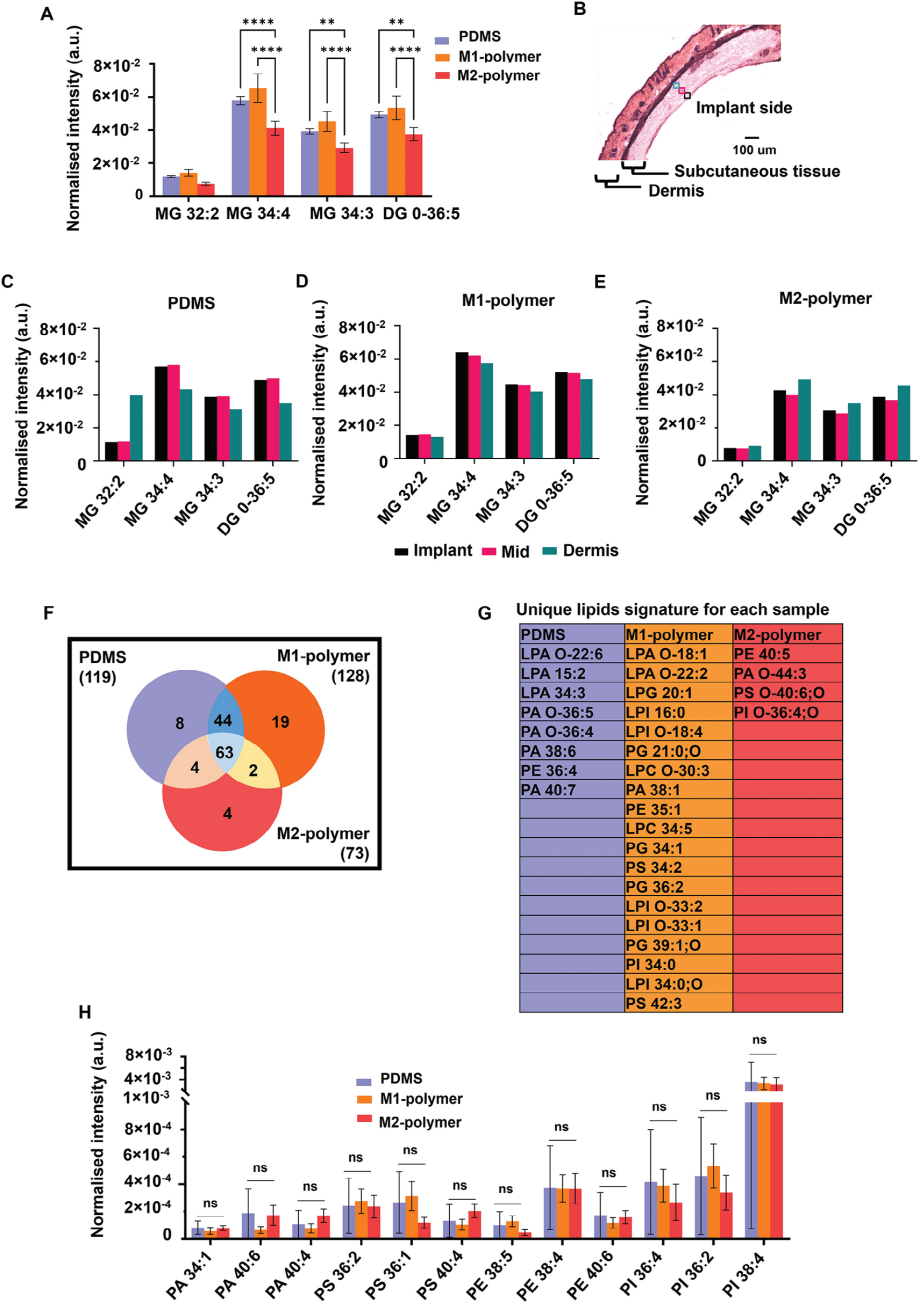
Relative quantification of characteristic lipid fragments detected by OrbiSIMS in positive and negative ion mode. A) The normalized intensity of four different glycerolipid species in each tissue sample. B) H&E stain image shows the three regions further away from the implant was analyzed, implant (black), mid‐point (pink), and dermis (sky blue). The normalized intensity of glycerolipid as a function of distance from the implant in each sample C) PDMS, D) M1‐polymer, and E) M2‐polymer. F) Venn diagram comparison of the number of phospholipids detected in 3 different tissue samples using 3D OrbiSIMS and unique lipid signature for each sample. G) The list of unique lipid signatures in each sample. H) Normalized intensity of phospholipids in three separate samples.

The relative intensity of the four most intense glycerol lipids correlated with the pro‐inflammatory polymer implants are compared between the three implants in **Figure** **3**A where statistical significance is found for the three most intense molecules. These observations are consistent with previous work that determined that glycerol lipid production influences immune cell activity and enhances inflammation.^[^ ^27^ ^]^

To investigate the glycerol lipid distribution as a function of distance from the implant in the subcutaneous adipose tissue, mass spectra were acquired from three different areas across the tissue section, ≈50 µm apart (next to the implant, mid‐point, and next to the dermis) as shown in Figure 3B. The glycerolipid intensities are presented for each implant in Figure 3C–E and in Table S1, Supporting Information. The M1‐inducing polymer implant was associated with a higher intensity of glycerolipid peaks at the implant surface compared to silicone and the M2‐inducing polymer‐coated catheters (Figure 3D). Combining this with the observation of FBR encapsulation thickness, this indicated that glycerolipids are increased in pro‐inflammatory tissue microenvironments associated with lower FBR.

It is known that lipid molecules have potent immunologic effects that can affect inflammation and fibrosis.^[^ ^28^ ^]^ A recent study by Schreib et al., reported that lipids were deposited by host cells on the surface of a biomaterial after implantation and that the types of lipids correlate with how the immune system reacts to the biomaterial.^[^ ^9^ ^]^ The anti‐inflammatory implants demonstrated significant enrichment of phospholipids hypothesized to be anti‐fibrotic at the explanted implant surface. Our observation of glycerolipids in the tissue above the anti‐fibrotic M1‐inducing samples in our study suggests that these two observations may be linked.

Glycerolipids are essential structural components of cell membranes, influencing membrane fluidity, ion exchange, and apoptotic signals and are the main long‐term energy‐storing molecules in mammalian cells and act as a second messenger signaling lipid.^[^ ^29^, ^30^, ^31^ ^]^ MG, DG, and TG are types of glycerolipids consisting of one, two, and three fatty acids respectively through an ester bond.^[^ ^32^ ^]^ It has been demonstrated that glycerolipid production influences immune cell activity and enhances inflammation.^[^ ^33^ ^]^ For example, DG functions as a second messenger that modulates the activation of protein kinase C (PKC), an enzyme that contributes to T‐cell activation and proliferation, hence preserving the integrity of the cell membrane.^[^ ^34^ ^]^ The accumulation of DG can lead to a state of lipotoxicity, which causes cell dysfunction and apoptosis and can also induce diabetes and cancer.^[^ ^35^ ^]^ The clear identification of glycerolipids in the tissue adjacent to M1‐inducing implants is a strong demonstration that the OrbiSIMS tissue metabolite profiling successfully detected metabolic tissue change.

Phospholipids were detected at lower ion intensities than the glycerol lipids, but those common and unique to the three tissues are compared in Figure 3F. A higher number of phospholipid compounds were detected from the tissue next to the M1 polymer than the uncoated silicone and the anti‐inflammatory polymer. A total of 128 lipid peaks were putatively identified in tissue near the pro‐inflammatory polymer, of which 63 lipid ions were common to the uncoated silicone and anti‐inflammatory polymers. In addition, there were four unique lipid compounds in tissue near the anti‐inflammatory polymer identified using the LIPID MAPS database (Table S2, Supporting Information). The list of unique phospholipid signatures in each sample is shown in Figure 3G indicating the greater number of species adjacent to the M1‐polymer tissue, however for 12 representative phosphatidic acid (PA), phosphatidylserine (PS), phosphatidylethanolamine (PE), and phosphatidylinositol (PI) assignments shown in Figure 3H, no statistically significant intensity differences were observed. (Table S3, Supporting Information). Recent research has focused on the various biological impacts of PA produced by activated macrophages and numerous other cells.^[^ ^29c,d^ ^]^ Particularly intriguing is the fact that PA functions as an intermediary messenger for several selective pro‐inflammatory targets. PA has been reported to protect lipopolysaccharide (LPS)‐induced septic mice by pharmacologic inhibition.^[^ ^29e^ ^]^ The greater number of phospholipid species is in general agreement with the SIMS study of Schreib et al^[^ ^9^ ^]^ where lower FBR implants correlated with phospholipids after 24 h of implantation.

The confirmation of the identity of some of our putative lipid assignments chosen since they were also seen in our single‐cell macrophage SIMS study^[^ ^26c^ ^]^ was achieved using tandem mass spectrometry (MS/MS) in the Orbitrap when analyzing the tissue section sample. We performed MS/MS confirming the negative ion assignments for the PA and PI as shown in the resulting spectra (Figure S4A–D, Supporting Information). Moreover, MS/MS confirmed the identity of the fragments from key mass ions in the tissue sample providing structural information on the key lipid species including the constituent fatty acid moieties, and lipid class.

**Figure 4:**
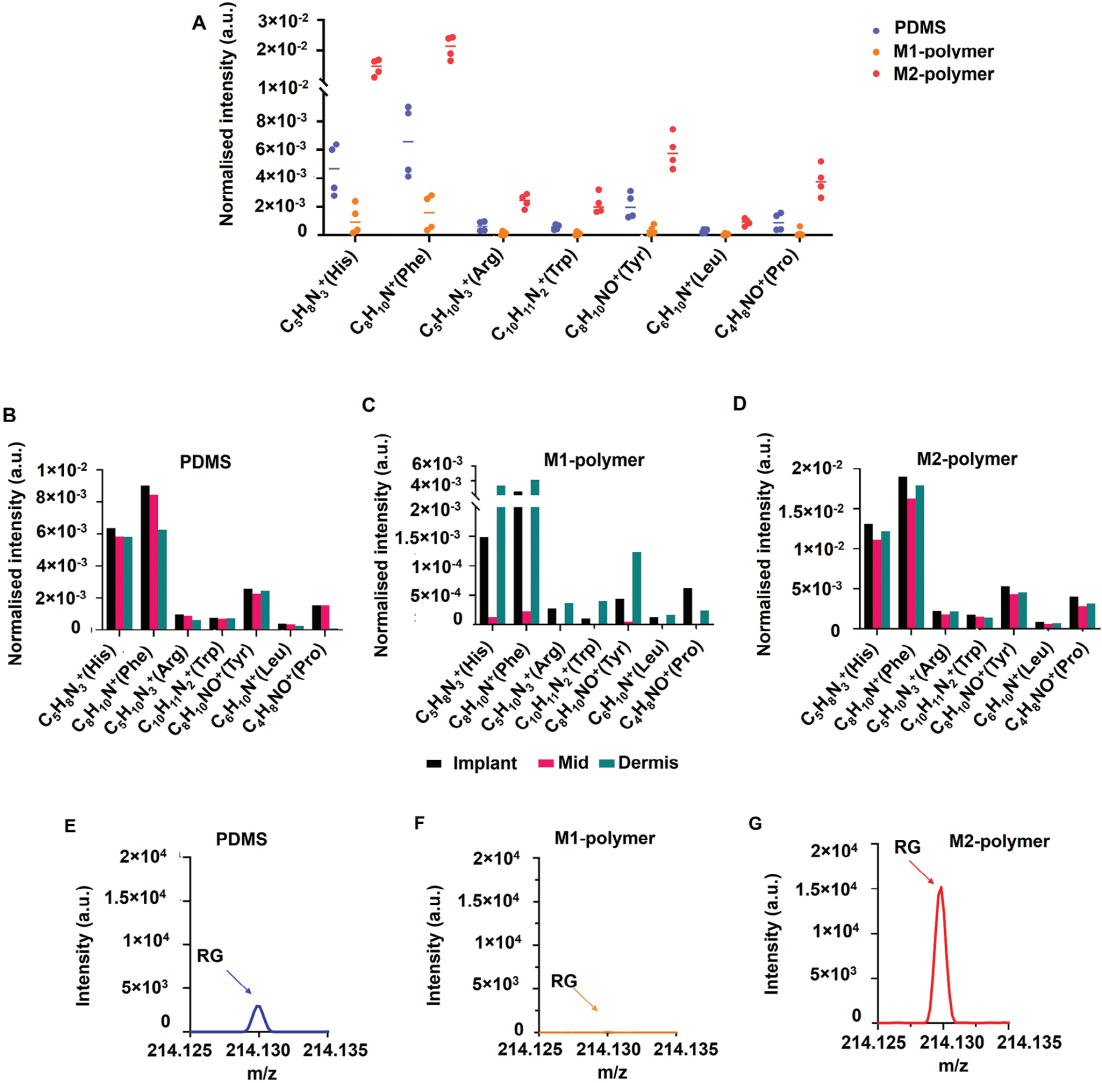
Characteristic amino acid fragments were detected in the tissue section in positive ion mode. A) The normalized intensity of amino acid fragments in each sample, PDMS (blue), M1‐polymer (orange), and M2‐polymer (red). The normalized intensity of amino acids further away from the implant in each sample B) PDMS, C) M1‐polymer, and D) M2‐polymer, the implant (black), mid‐point (pink), and dermis (sky blue). E‐G) The spectrum of amino acids with RG sequences from each tissue sample.

Amino acid ion intensities were observed to be higher for M2‐inducing polymer implants in Figure 2B. These were quantified in **Figure** **4**A, where large intensity differences are observed between the three implant polymers, this is consistent with the collagenous layer preferentially around the M2‐ polymer implant (Figure S1C, Supporting Information). A total of 52 amino acid fragments were assigned from the tissue sample (Table S4, Supporting Information). Evaluating the ion intensity versus distance from the implant in Figure 4B–D, it is apparent that the greatest variance between the three positions is seen in the tissue exposed to the M1 polymer implant, with the highest amino acid ion intensities seen at the subcutaneous adipose tissue abutting the interface with the implant or at the dermis interface.

The amino acid intensities are, more similar with distance for the uncoated silicone and M2 polymer implants (Figure 4C and D). The characteristic amino acid ions observed are consistent with fragmentation from proteins. Kotowska et al.,^[^ ^26a^ ^]^ gathered lysozyme fragments in a spectrum from a protein monolayer sample, with the Arginine‐Glycine (RG) amino acid pairs of lysozyme detected at m/z 214.1295 [C_8_H_16_N_5_O_2_]^+^. This protein fragment was also seen from the tissue samples and at similar relative intensities between samples, suggesting that these mono amino acid signals are fragments from proteins, and are not from free amino acids (Figure 4E–G; Table S5, Supporting Information).

### 2.3 Comparison of Tissue and Single Cell Metabolites

**Figure 5:**
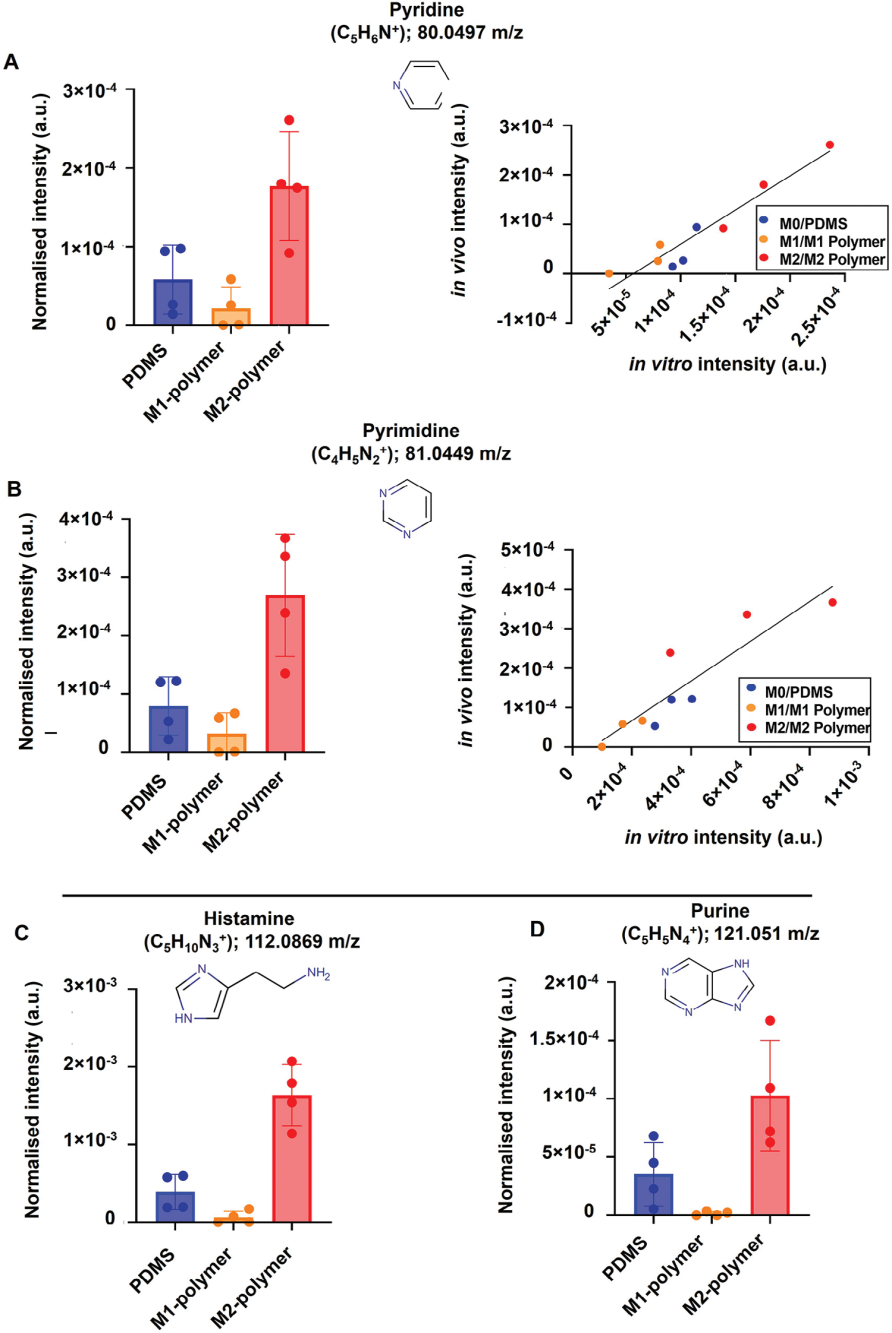
A) Metabolites detected from in vivo tissue were significantly affected by M1 and M2‐polymers. B) Comparing the compounds pyridine and pyrimidine from in vivo tissue samples and single‐cell macrophage analysis shows simple linear relationships for pyridine and pyrimidine.^[^ ^39^ ^]^ C) Histamine from in vivo and D) Purine from in vivo are found predominantly in tissue adjacent to M2‐polymer or PDMS.

A range of non‐lipid metabolites were detected by 3D OrbiSIMS in the analysis of individual macrophage cells in vitro preferentially expressed by M1, M2, or M0 cells, identified using the Human Metabolome Database.^[^ ^26c^ ^]^ Pyridine (C_5_H_6_N^+^) and pyrimidine (C_4_H_5_N_2_^+^) had comparatively high ion intensities after M2 polarisation. This is consistent with our findings that pyridine moieties are intense in tissue adjacent to M2‐polymer while being much lower in intensity for tissue adjacent to M1‐polymer implants (**Figure** **5**A,B). The single cell in vitro intensity is plotted versus the in vivo tissue intensity of pyridine and pyrimidine and was shown to be highly correlative (simple linear regression curves: R^2^ = 0.91 for pyridine; R^2^ = 0.82 for pyrimidine), indicating that in vitro cell studies can semi‐quantitatively predict in vivo pyridine and pyrimidine intensities. These correlations between single‐cell analysis and tissue stimulated by implanted polymers give support to the use of molecular characterization to link in vitro and in vivo performance and its role in probing underpinning molecular mechanistic understanding.

Furthermore, two molecules, histamine (C_5_H_10_N_3_^+^) and purine (C_5_H_5_N_4_^+^), were detected strongly from tissue abutting the M2 polymer and not at all from M1 polymer (Figure 5C,D; Table S6, Supporting Information). Both compounds are connected to anti‐inflammatory cellular reactions and so are consistent with the M2‐inducing polymers exerting influence in the local tissue environment. Histamine can promote wound healing in skin lesions, inhibit tumor growth, and modulate inflammation in models of colitis and experimental autoimmune encephalomyelitis (EAE).^[^ ^36^ ^]^ Purine, a common substrate in living organisms, is essential for cellular proliferation and a key regulator of the immune system. Multiple enzymes carefully regulate the purine de novo and salvage pathways, and malfunction in these enzymes results in excessive cell proliferation and immunological imbalance, which leads to tumor growth.^[^ ^37^ ^]^ Furthermore, purine has antioxidant and anti‐inflammatory properties, as well as a role in cell energy homeostasis.^[^ ^38^ ^]^

### 2.4 Imaging of Metabolites in the Tissue Abutting the Implants

**Figure 6:**
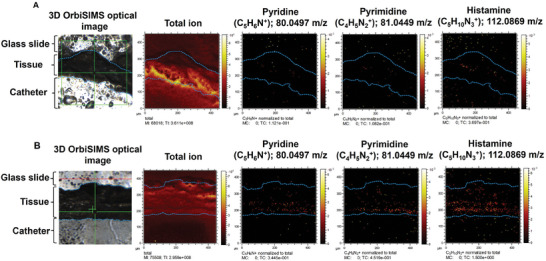
Chemical imaging of tissue sample. A,B) Ion images of M1‐plymer and M2‐polymer were acquired (area of 450 × 450 µm), including pyridine, pyrimidine, and histamine metabolite which are divided by total intensity.

**Figure 7:**
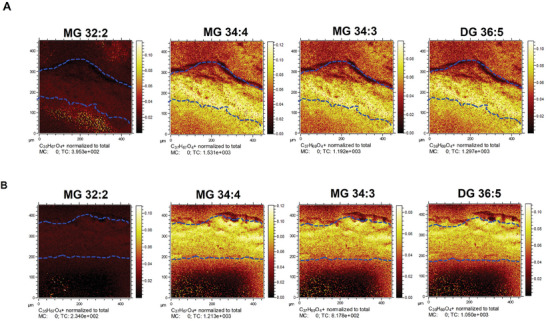
Chemical imaging of tissue sample. (A,B) Ion images of the sum of glycerolipids in M1‐polymer and M2‐polymer were acquired (area of 450 × 450 µm), including MG and DG which are divided by total intensity. The blue lines denoted the upper and lower boundaries of the tissue, with the catheter at the bottom of the image.

Using the 3D OrbiSIMS in imaging mode, the distribution of metabolites of interest from the above spectral analyses was produced as shown in **Figure** **6**A,B from a 450 × 450 µm area of the subcutaneous adipose tissue interface adjacent to the implant. It is interesting that the M2 metabolites were located in bands near the interface between tissue and implant. As expected from the spectral analysis (Figure 5), they were seen more strongly in the M2‐polymer image for pyridine, pyrimidine, and histamine. The glycerides appear intense and uniformly distributed. (**Figure** **7**A,B).

Representative molecular ions of the phospholipid classes PA, PS, PE, and PI are shown in Figure S3, Supporting Information. The PI ions were the most intense compared to the other phospholipid species and the distribution of each ion seemed similar. We mapped the PI 38:4, [C_47_H_82_O_13_P]^−^ and [C_6_H_10_PO_8_]^−^ ion corresponding to the PI head group, in Figure S4, Supporting Information, and two fatty acids are represented by FA 18:0 [C_18_H_35_O_2_]^−^ peak at m/z 283.2642 and FA 20:0 [C_20_H_31_O_2_]^−^ peak at m/z 303.2327. We also mapped the nuclear marker and overlayed it with the PI 34:4 marker as shown in Figure S3, Supporting Information. The ion contribution of nuclear marker [HP_2_O_6_]^−^ at m/z 158.9056 is mapped in blue, and PI 34:4 at m/z 885.5500 is mapped in red. The nuclear marker intensity distribution is more closely correlated to the phospholipids than the glycerides, suggesting that a proportion of these lower‐intensity species are generated from cells.

## 3 Conclusion

We report a new label‐free direct analysis strategy able to provide molecular insight into the host‐implant interface using 3D OrbiSIMS. This study provides a detailed molecular characterization of tissue sections, allowing information on the distribution of metabolites, lipids, and protein‐derived amino acids. Silicone catheter sections coated with different immune‐instructive polymers were used as example medical devices in a rodent model of FBR. Novel M1‐ and M2‐inducing polymer‐coated implants produced distinct tissue metabolite profiles revealed by 3D OrbiSIMS. Interestingly, glycerolipids seem to be more abundant in tissue adjacent to pro‐inflammatory surfaces. For some metabolites, the in vivo ion intensities were found to correlate with a single cell in vitro analysis of polarised macrophages, highlighting the power of this approach in elucidating cell responses in a complex biological context. A better understanding of immunometabolism has started to provide new insights into many pathologies as well as the immune system's role in maintaining tissue homeostasis. Combining powerful metabolic imaging techniques and biomaterial design could be transformative in enabling the design of novel bio‐instructive materials that present positive interactions with the immune system to induce a pro‐healing macrophage response following implantation.

## 4 Experimental Section

### Implant Sample Preparation

Clinical‐grade silicone rubber catheters with a 13 mm external diameter were cut to a length of 5 mm (cylinder shape). To secure in an automated dip coating rig, micro‐lance needles were inserted into the catheter wall and clamped. The catheters were prepared by dipping them into Nusil MED1‐161 silicone primer, which was made up of tetrapropylsilicate and tetra (two‐methoxyethoxy) in 50% v/v acetone and withdrawing rate of 1 mm min^−1^ for 30 s. They were then dried at room temperature for 2 min. MED1‐161 coated catheters were dip‐coated into a copolymer solution of each of the polymers (5% w/v) in dichloromethane with a dipping and withdrawing rate of 1 mm min^−1^ for 30 s twice. Copolymers were synthesized via a thermal free radical polymerization method, purified by precipitation into an excess of methanol, and then characterized by Nuclear magnetic resonance spectroscpy (NMR) and Gel Permeation Chromatography (GPC). The polymers used had previously been identified to polarize macrophages in vitro and modulate the FBR in vivo: pCHMA‐DMAEMA (referred to as M1 polymer) which induce pro‐inflammatory macrophage or pCHMA‐iDMA polymer (referred to as M2 polymer) which induce anti‐inflammatory macrophage phenotype.^[^ ^11^ ^]^ Coated catheters were dried overnight at room temperature and then dried in a vacuum at 50 °C (<0.3 mbar) for 7 days to remove solvent. The chemical structure of the monomers and copolymers pCHMA‐co‐DMAEMA and pCHMA‐co‐iDMA are presented in Table S7, Supporting Information.

### In Vivo Models

In vivo studies were approved by the University of Nottingham Animal Welfare and Ethical Review Board and carried out in accordance with Home Office authorization under project license number PP5768261. Female BALB/c mice, 19–22 g were used in these studies and were housed in individually ventilated cages (IVCs) under a 12 h light cycle, with access to food and water ad libitum. The weight and clinical condition of the mice were monitored daily. UV light was used for 20 min to sterilize the catheter segments prior to implantation. All segments were implanted subcutaneously into mice for 28 days, 3 mice/each polymer. At the end of the animal studies, on day 28, mice were humanely sacrificed by CO_2_ euthanasia. The polymer identity was blinded to the researchers until the end of the data quantification.

### Histological Analysis

The catheter segments and surrounding skin after 28 days of implantation were cut to ≈5.5 × 5.5 cm and were embedded in an optimal cutting temperature compound (OCT). Following embedding, the tissue was placed in a cryostat chamber at −20 °C and sliced into 15 µm thick sections (CM1850, Leica microsystems). The FBR to the polymer coatings was assessed by staining with haematoxylin and eosin (H&E) in Table S8, Supporting Information, and Masson's trichrome (MTC) using optimized protocols contained in Tables S9, S10, Supporting Information respectively. Following H&E staining, images were recorded using an Axioplan microscope (Zeiss) with a 40X objective to count the number of macrophages and neutrophils (field of view 100 × 100 um) N = 2 and *n* = 3. With MTC staining, each sample was captured at 10× magnification, and the thickness of the collagen was measured by taking four measurements from the top to the bottom of the distinct layer: one at the top, one down, and the other two further away cross of the images of each sample, N = 3 and *n* = 1.

### Macrophage Phenotype Analysis

The method used was taken from Rostam et al.^[^ ^11^ ^]^ Briefly, tissue sections were stained to identify the macrophage phenotype at the catheter‐tissue interface. This was carried out using the pro‐inflammatory marker inducible iNOS and the anti‐inflammatory marker Arg‐1. The processing of the macrophage phenotype is shown in Table S4, Supporting Information.^[^ ^11^ ^]^ The stained cells were imaged with a Zeiss LSM880C confocal microscope, and any background fluorescence was subtracted using ImageJ. The mean raw fluorescence intensity density of the region of interest around the foreign body site was used to measure the sum of all pixels in the given area and at least five different fields of view were randomly examined in each tissue section. A signal‐to‐noise ratio (SNR) of two as the detection threshold for fluorescence intensity measurements by ImageJ software was used. The fluorescence intensity ratio of M2 macrophages to M1 macrophages was calculated for each region.

### OrbiSIMS Analysis

After tissue sectioning, the slices of tissue supported on slides were gently washed three times with cold distilled water for 30 s, one time with cold 70% ethanol for 30 s, and then 3 times with 1 mL of 150 mM ammonium formate solution for 30 s to remove salts which can decrease the sensitivity of molecules in SIMS by signal suppression.^[^ ^40^ ^]^ Tissue slides were plunged frozen in liquid nitrogen and then freeze‐dried for 12 h to remove water whilst retaining some degree of 3D structure. The samples were subsequently stored in a microscope slide box container at −80 °C until analysis. Prior to OrbiSIMS analysis, the sample was warmed to room temperature without opening and then mounted onto the instrument sample holder and loaded into the 3D OrbiSIMS for analysis. 3D OrbiSIMS analysis was conducted with a Hybrid SIMS instrument (IONTOF, Germany) using Mode 4 which comprised a single Ar_3000_^+^ primary ion beam of energy of 20 keV a duty cycle of 4.4% and continuous GCIB current of 2.3 A, over an area of 100 × 100 µm with crater‐size 180.0 × 180.0 µm collecting data using the OrbiTrap analyzer in the mass range of *m/z* 75–1125. The electron flood gun was operated with an energy of 21 eV and an extraction bias of 20 V. for charge compensation. The pressure in the main chamber was maintained at 8.9 × 10^−7^ mbar using argon gas flooding. The OrbitrapTM cycle time was set to 200 µs. The Orbitrap analyzer was operated in positive and negative ion mode at the mass resolving power setting of 240000 (at *m/z* 200). The secondary ion injection time was 500 ms, and the total ion dose per measurement was 5.21 × 10^10^ ions/cm^2^. Adjacent areas on the tissue samples were analyzed, four regions surrounding the implant region (catheter‐tissue interface) per one tissue section slide and three regions further away the implant (next to the implant, mid‐point, and next to the dermis) were consumed with both positive and negative polarity.

One 3D OrbiSIMS image using the 20  keV Ar_3000_^+^ analysis beam with a 2 µm diameter probe was acquired. The 20 µm analysis beam was configured as described in the spectra acquisition section. The pixel size 3 µm imaging beam duty cycle was set to 37.7% and GCIB current was 2.3 A. The images were run on the area of 450 ×  450 µm using random raster mode. The cycle time was set to 400 µs. Argon gas flooding was in operation; to aid charge compensation, pressure in the main chamber was maintained at 9.0 × 10^−7^ mbar using argon gas flooding. The images were collected in negative polarity, in a mass range 75–1125 *m/z*. The injection time was set to 500 ms, and the total ion dose per measurement was 1.61 × 10^13^. Mass‐resolving power was set to 240000 at 200 *m/z*. All data analysis was carried out using Surface Lab 7.1 software (IONTOF GmbH).

### Principal Component Analysis (PCA)

The 3D OrbiSIMS spectra contained many secondary ions. Principal component analysis was applied to the data set to provide unbiased identification of the differences between each tissue sample. Spectra of all tissue samples were acquired by accumulating data from a single area, four areas of each sample were acquired, with each normalized to their respective total ion count in SurfaceLab 7 software. A peak list was constructed, containing the ions above the minimum ion count intensity, which was determined in each case as being greater than assigned noise signals (1428 peaks in the positive polarity spectra). Multivariate analysis of 3D OrbiSIMS results was done in simsMVA (https://mvatools.com/), using Matlab.^[^ ^41^ ^]^ The peak list was normalized to the total ion count and applied to four regions of interest on all samples. The data was pre‐processed by Poisson scaling and mean centering. PCA was run in algorithm mode, retaining all principal components.

### Data Processing and Metabolites Identification

Peak assignments for each sample were created by IonTOF SurfaceLab 7. Peak lists of secondary mass ions and secondary intensity from the software were exported and then imported into the LIPIDMAPS database to identify the lipid species. For metabolite results, 3D OrbiSIMS spectra were exported as .txt files and then searched against the Human Metabolome Database with a 5 ppm mass tolerance for putative annotation.

## Conflict of Interest

The authors declare no conflict of interest.

## Supporting information

Supporting Information

## Data Availability

The data that support the findings of this study are available in the supplementary material of this article.
