# Supplementary material for: Spatially Resolved Molecular Analysis of Host Response to Medical Device Implantation Using the 3D OrbiSIMS Highlights a Critical Role for Lipids
Source: Adv Sci (Weinh). 2024 Feb 14;11(15):2306000. doi: 10.1002/advs.202306000 (PMC11022720; doi:10.1002/advs.202306000)
Supplement: Supplementary file 1 — Supporting Information [file ADVS-11-2306000-s001.pdf]

ADVANCED SCIENCE
Open Access

Supporting Information

for *Adv. Sci.*, DOI 10.1002/advs.202306000

Spatially Resolved Molecular Analysis of Host Response to Medical Device Implantation Using the 3D OrbiSIMS Highlights a Critical Role for Lipids

*Waraporn Suvannapruk, Leanne E Fisher, Jeni C Luckett, Max K Edney, Anna M Kotowska, Dong-Hyun Kim, David J Scurr, Amir M Ghaemmaghami and Morgan R Alexander**

Supporting Information

Spatially resolved molecular analysis of host response to medical device implantation using the 3D OrbiSIMS

Waraporn Suvannapruk[a1], Leanne E Fisher[a], Jeni C Luckett[b], Max K Edney[c], Anna Kotowska[a], Dong-Hyun Kim[a], David J Scurr[a], Amir M Ghaemmaghami[d] and Morgan R Alexander[a*]

[a] Advanced Materials and Healthcare Technologies Division, School of Pharmacy, University of Nottingham, University Park Nottingham, NG7 2RD, United Kingdom.

[b] School of Life Sciences, Faculty of Medicine and Health Science, University of Nottingham, University Park Nottingham, NG7 2RD, United Kingdom.

[c] Department of Chemical and Environmental Engineering, Faculty of Engineering, University of Nottingham, University Park Nottingham, NG7 2RD, United Kingdom.

[d] Immunology & Immuno-bioengineering Group, School of Life Sciences, Faculty of Medicine and Health Sciences, University of Nottingham, University Park Nottingham, NG7 2RD, United Kingdom.

[1] National Metal and Materials Technology Center (MTEC), National Science and Technology Development Agency (NSTDA), 111 Phahonyothin Road, Khlong Nueng, Khlong Luang, Pathum Thani 12120, Thailand.

Corresponding Author: *E-mail: morgan.alexander@nottingham.ac.uk

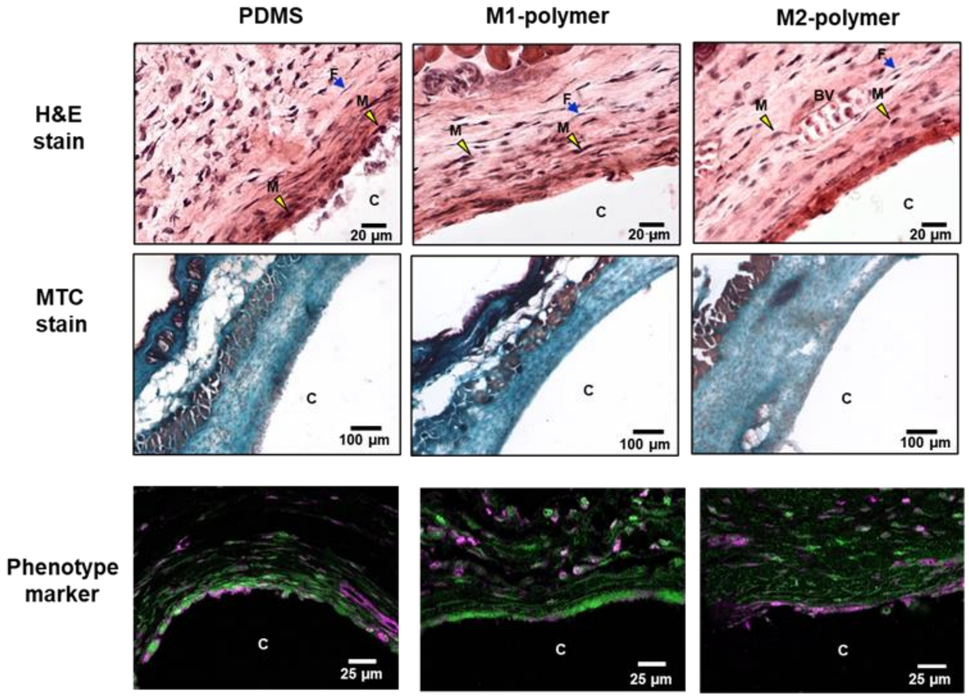
PDMS
M1-polymer
M2-polymer
H&E stain
MTC stain
Phenotype marker
F
M
BV
C
20 µm
100 µm
25 µm

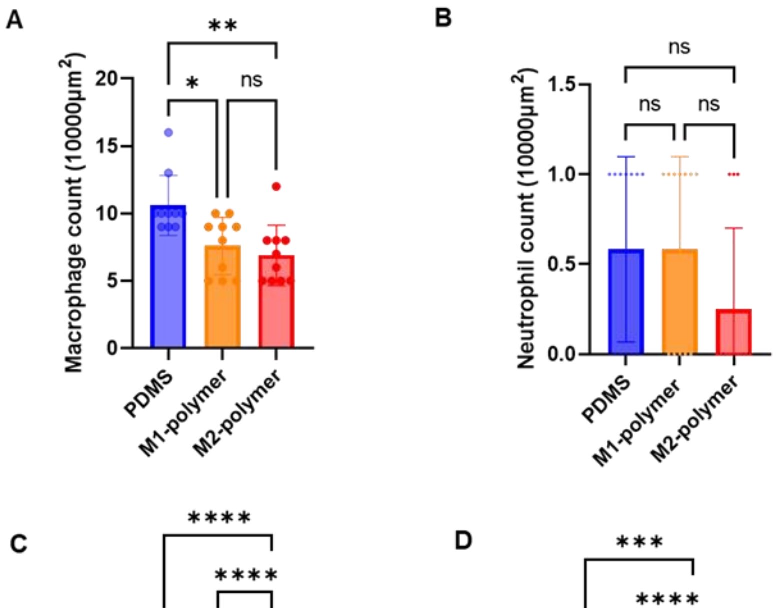
A
B
C
D
Macrophage count (10000µm^2)
Neutrophil count (10000µm^2)
PDMS
M1-polymer
M2-polymer
**
*
ns
***
****

Figure. S1. Histological analysis of tissue sections following 28 days implantation of polymer coated catheter sections in a murine model of foreign body response. Representative H&E staining images, showing a well-defined inflammatory reaction fibroblast (F), blood vessels (BV), macrophage (M) and catheter (C) captured at × 40 magnification the scale bar = 20 μm. MTC staining image of each tissue section slide for identifying collagen thickness, captured at × 10 magnification. Scale bar = 100 μm. (A-C) show the infiltration count of macrophages, neutrophils and collagen thickness from the site surrounding the foreign body. All data are presented as the mean with ±s.d (N=2, n=3). Significance was calculated by one-way ANOVA with Tukey's post-hoc analysis: *p<0.05, ** p<0.01, *** p<0.001. (D) Representative images show tissue section stained for the M1 marker iNOS in green and M2 marker arginase1 in magenta and C represents the catheter site. (Images were acquired on confocal). Scale bar = 25 μm. (D) The ratio of M2-like macrophages to M1-like macrophages for each polymer. All data are presented as the mean with ±s.d (N = 2 and n = 5). Significance was calculated by one-way ANOVA with Tukey's post-hoc analysis: *** <0.0001

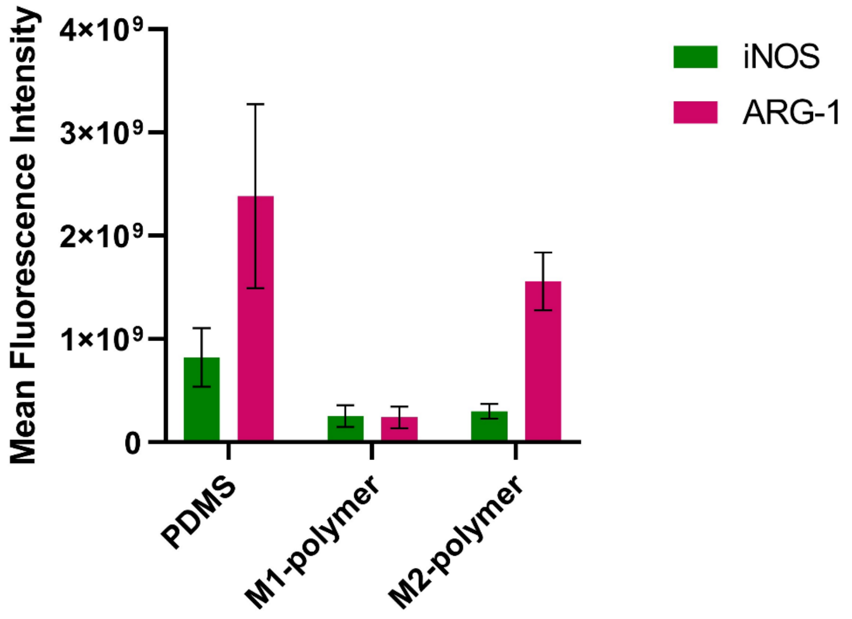

Figure. S2. The mean fluorescescence intensity of iNOS and ARG-1 expression in tissue images. M2-polymer shown high level of the Arg-1 expression.

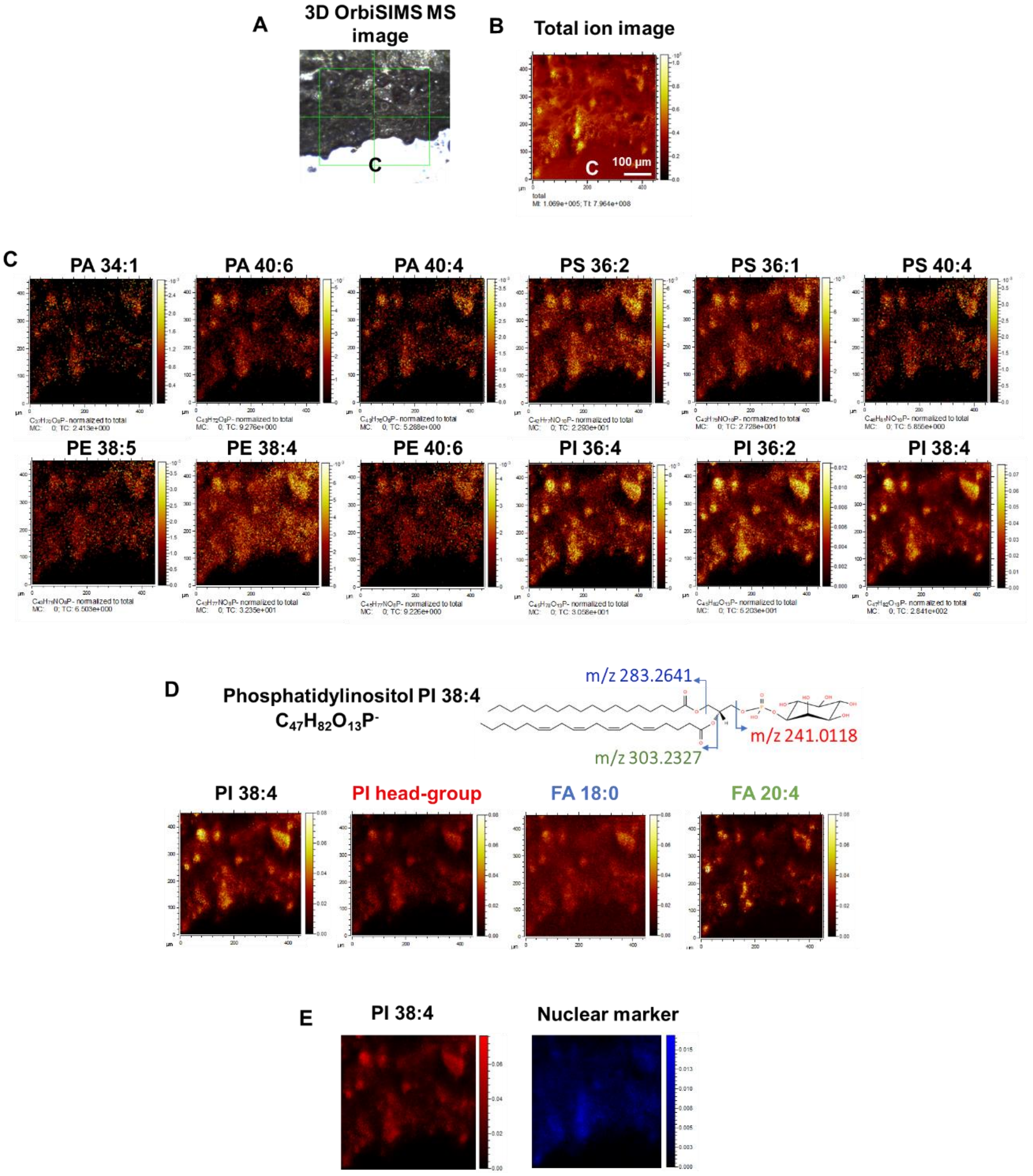

A
3D OrbiSIMS MS image
B
Total ion image
100 µm
C
PA 34:1
PA 40:6
PA 40:4
PS 36:2
PS 36:1
PS 40:4
PE 38:5
PE 38:4
PE 40:6
PI 36:4
PI 36:2
PI 38:4
D
Phosphatidylinositol PI 38:4
C47H82O13P-
m/z 283.2641
m/z 241.0118
m/z 303.2327
PI 38:4
PI head-group
FA 18:0
FA 20:4
E
PI 38:4
Nuclear marker

Figure. S3. Chemical imaging of tissue sample. (A) View of the area where OrbiSIMS optical ion images were acquired (area of 450 µm × 450 µm). (B–D) 3D OrbiSIMS ion images were recorded in the negative ion mode, (B) total ion image, (C) ion image of the sum of phospholipid specie ions including PA, PS, PE and PI which are divided by total intensity. (D) The main ion of PI (38:4), showing the contribution of PI (38:4) ion are the signature fragments of PI head group, two fatty acids fragments (FA 18:0 and FA 20:4) and (E) RGB ion images showing nuclear marker (blue) and PA 34:4 (red).

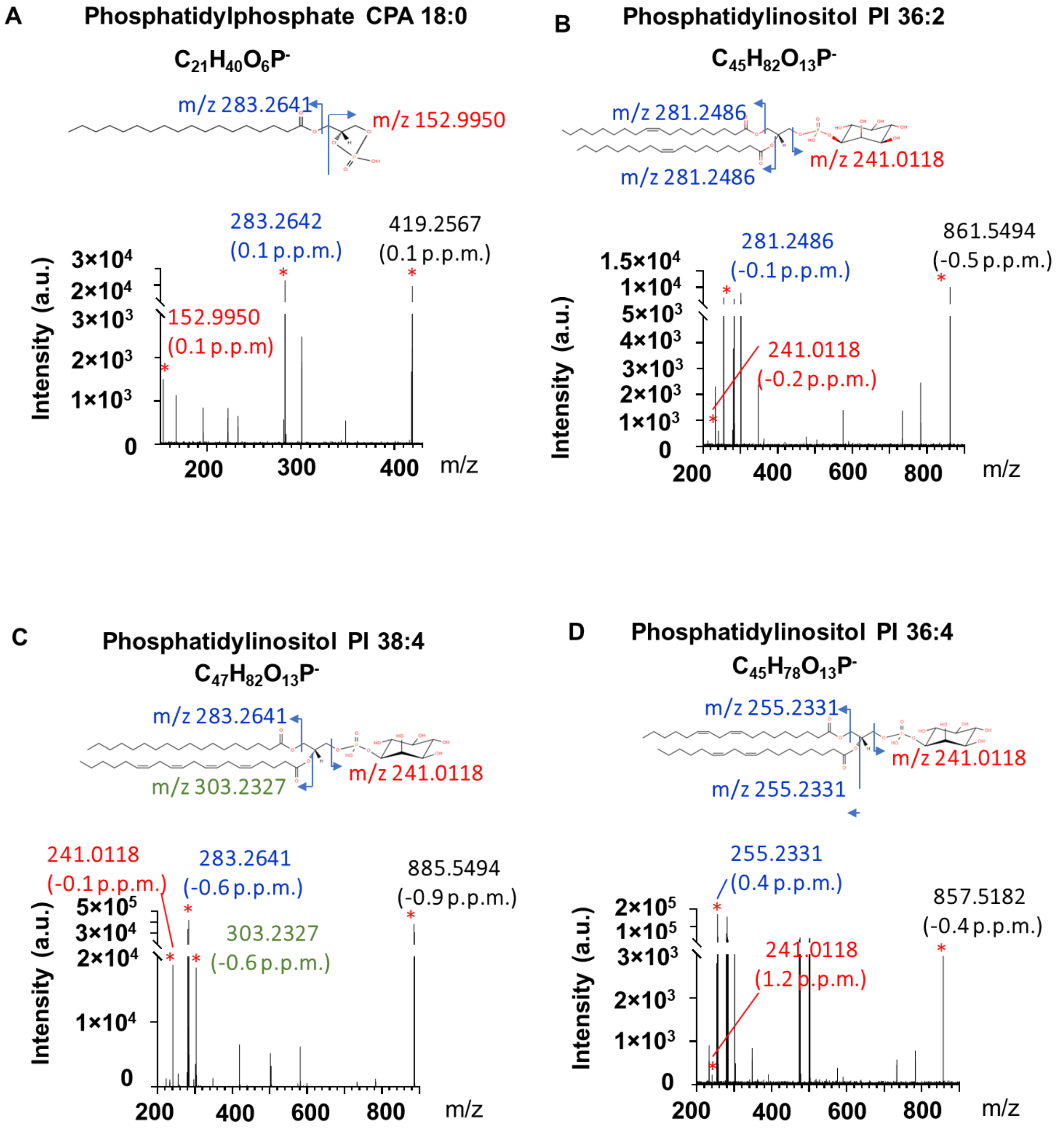
A
Phosphatidylphosphate CPA 18:0
C21H40O6P-
m/z 283.2641
m/z 152.9950
283.2642
(0.1 p.p.m.)
419.2567
(0.1 p.p.m.)
152.9950
(0.1 p.p.m)
Intensity (a.u.)
3×10^4
2×10^4
3×10^3
2×10^3
1×10^3
0
200
300
400
m/z
B
Phosphatidylinositol PI 36:2
C45H82O13P-
m/z 281.2486
m/z 241.0118
m/z 281.2486
281.2486
(-0.1 p.p.m.)
861.5494
(-0.5 p.p.m.)
241.0118
(-0.2 p.p.m.)
Intensity (a.u.)
1.5×10^4
1×10^4
5×10^3
4×10^3
3×10^3
2×10^3
1×10^3
0
200
400
600
800
m/z
C
Phosphatidylinositol PI 38:4
C47H82O13P-
m/z 283.2641
m/z 241.0118
m/z 303.2327
241.0118
(-0.1 p.p.m.)
283.2641
(-0.6 p.p.m.)
885.5494
(-0.9 p.p.m.)
303.2327
(-0.6 p.p.m.)
Intensity (a.u.)
5×10^5
3×10^4
2×10^4
1×10^4
0
200
400
600
800
m/z
D
Phosphatidylinositol PI 36:4
C45H78O13P-
m/z 255.2331
m/z 241.0118
m/z 255.2331
255.2331
(0.4 p.p.m.)
857.5182
(-0.4 p.p.m.)
241.0118
(1.2 p.p.m.)
Intensity (a.u.)
2×10^5
1×10^5
3×10^3
2×10^3
1×10^3
0
200
400
600
800
m/z

Figure. S4. MS/MS product spectrum of lipids [M-H]-. (A) The structure of CPA (18:0) at m/z 419.2517 was confirmed based on the detection of the PA head-group ions, [C6H10PO8]- at m/z 241.0, and fatty acid moieties from these lipids are F18:0, [C18H35O2]- at m/z 283.2642. (B) The precursor PI (36:2) ion is the signature fragments of PI headgroup, [C6H10PO8]- and two FA18:1 fatty acid moieties are represented by [C18H33O2]-. and other MS/MS of PI (38:4) and (36:4) lipids are reported in Figure C and D).

Table S1. Glycerolipid fragments in 3D OrbiSIMS at surrounding implants in each sample PDMS, polymer M1-polymer and polymer M2-polymer. Monoglycerides (MG), diglycerides (DG)

Mass m/z	Assignment	Glycerolipid	Average normalised Intensity (4 areas)	Average p.p.m (4 areas)
**PDMS**				
551.504	$C_{35}H_{67}O_4^+$	MG 32:2	1.18×10^{-2}	1
575.5038	$C_{37}H_{67}O_4^+$	MG 34:4	5.79×10^{-2}	0.6
577.5193	$C_{37}H_{69}O_4^+$	MG 34:3	3.92×10^{-2}	0.4
601.5195	$C_{39}H_{69}O_4^+$	DG 0-36:5	4.94×10^{-2}	0.6
**M1-polymer**				
551.504	$C_{35}H_{67}O_4^+$	MG 32:2	1.42×10^{-2}	0.9
575.5038	$C_{37}H_{67}O_4^+$	MG 34:4	6.53×10^{-2}	0.8
577.5193	$C_{37}H_{69}O_4^+$	MG 34:3	4.53×10^{-2}	0.7

601.5195	$C_{39}H_{69}O_4^+$	DG 0-36:5	5.35×10^{-2}	0.8
**M2-polymer**				
551.504	$C_{35}H_{67}O_4^+$	MG 32:2	7.50×10^{-3}	0.6
575.5038	$C_{37}H_{67}O_4^+$	MG 34:4	3.83×10^{-2}	0.3
577.5193	$C_{37}H_{69}O_4^+$	MG 34:3	2.93×10^{-2}	0.1
601.5195	$C_{39}H_{69}O_4^+$	DG 0-36:5	3.75×10^{-2}	0.3

Table S2. Show unique lipids signature for each phenotype; 8 unique for PDMS, 16 unique for M1-polymer and 4 unique for M2-polymer.

**Mass *m/z***	**Formula [M-H]$^-$**	**Name**
**PDMS**		
467.2567	$C_{25}H_{41}O_6P^-$	LPA O-22:6
476.2784	$C_{23}H_{44}NO_7P^-$	LPA 15:2
655.4709	$C_{37}H_{69}O_7P^-$	LPA 34:3
679.4712	$C_{39}H_{69}O_7P^-$	PA O-36:5
681.4867	$C_{39}H_{71}O_7P^-$	PA O-36:4

719.4662	$C_{41}H_{69}O_8P^-$	PA 38:6
738.5083	$C_{41}H_{74}NO_8P^-$	PE 36:4
745.4819	$C_{43}H_{71}O_8P^-$	PA 40:7
**M2-polymer**		
795.5743	$C_{45}H_{85}NO_8P^-$	LPI O-33:0
795.6271	$C_{47}H_{89}O_7P^-$	PA O-44:3
836.5446	$C_{46}H_{80}NO_{10}P^-$	PS O-40:6;O
859.534	$C_{45}H_{81}O_{13}P^-$	PI O-36:4;O
**M1-polymer**		
421.2728	$C_{21}H_{43}O_6P^-$	LPA O-18:1
475.3195	$C_{25}H_{49}O_6P^-$	LPA O-22:2
537.32	$C_{26}H_{51}O_9P^-$	LPG 20:1
571.2891	$C_{25}H_{49}O_{12}P^-$	LPI 16:0
577.2787	$C_{27}H_{47}O_{11}P^-$	LPI O-18:4
585.3048	$C_{26}H_{51}O_{12}P^-$	PG 21:0;O
670.5186	$C_{38}H_{74}NO_6P^-$	LPC O-30:3
729.5445	$C_{41}H_{79}O_8P^-$	PA 38:1
730.5395	$C_{40}H_{78}NO_8P^-$	PE 35:1

736.5294	$C_{42}H_{76}NO_7P^-$	LPC 34:5
747.5172	$C_{40}H_{77}O_{10}P^-$	PG 34:1
758.4981	$C_{40}H_{74}NO_{10}P^-$	PS 34:2
773.533	$C_{42}H_{79}O_{10}P^-$	PG 36:2
791.5433	$C_{42}H_{81}O_{11}P^-$	LPI O-33:2
793.559	$C_{42}H_{83}O_{11}P^-$	LPI O-33:1
833.5917	$C_{45}H_{87}O_{11}P^-$	PG 39:1;O
837.5489	$C_{43}H_{83}O_{13}P^-$	PI 34:0
839.5643	$C_{43}H_{85}O_{13}P^-$	LPI 34:0;O
868.608	$C_{48}H_{88}NO_{10}P^-$	PS 42:3

Table S3. Targeted phospholipid analysis in 3D OrbiSIMS spectra.

**Mass *m/z***	**Assignment [M-H]$^-$**	**Phospholipids**	**Area1**		**Area 2**		**Area 3**		**Area4**	
			**Mass error p.p.m**	**Norm. intensity**	**Mass error p.p.m**	**Norm. intensity**	**Mass error p.p.m**	**Norm. intensity**	**Mas s error**	**Norm. intensity**

									p.p.m	
**PDMS**										
673.4821	$C_{37}H_{71}O_8P$	PA 34:1	0.3	1.54×10^{-4}	-0.1	5.52×10^{-5}	0.8	5.66×10^{-05}	1.0	5.79×10^{-05}
747.498	$C_{43}H_{73}O_8P$	PA 40:6	0.7	4.57×10^{-4}	0.3	7.92×10^{-5}	1.2	9.79×10^{-05}	0.8	1.10×10^{-04}
751.5291	$C_{43}H_{77}O_8P$	PA 40:4	0.3	2.57×10^{-4}	0	4.07×10^{-5}	0.9	5.80×10^{-05}	0.7	7.03×10^{-05}
786.5294	$C_{42}H_{78}NO_{10}P$	PS 36:2	0.4	5.42×10^{-4}	-0.2	1.18×10^{-4}	0.9	1.40×10^{-04}	0.8	1.65×10^{-04}
788.5451	$C_{42}H_{80}NO_{10}P$	PS 36:1	0.5	6.03×10^{-4}	-0.1	1.37×10^{-4}	1.3	1.55×10^{-04}	1.0	1.69×10^{-04}
838.5609	$C_{46}H_{82}NO_{10}P$	PS 40:4	0.6	3.10×10^{-4}	0.2	5.82×10^{-5}	1.2	7.27×10^{-05}	1.0	8.50×10^{-05}
764.5244	$C_{43}H_{76}NO_8P$	PE 38:5	0.4	2.47×10^{-4}	0	4.27×10^{-5}	1.1	5.37×10^{-05}	0.8	5.98×10^{-05}
766.5401	$C_{43}H_{78}NO_8P$	PE 38:4	0.4	8.32×10^{-4}	0.1	1.81×10^{-4}	1.1	2.31×10^{-04}	0.7	2.49×10^{-04}
790.5402	$C_{45}H_{78}NO_8P$	PE 40:6	0.4	4.20×10^{-4}	0	7.67×10^{-5}	0.9	8.64×10^{-05}	0.7	9.93×10^{-05}
857.5196	$C_{45}H_{79}O_{13}P$	PI 36:4	0.5	0.87×10^{-4}	0.2	1.79×10^{-4}	1.2	2.28×10^{-04}	1.0	2.66×10^{-04}
861.5511	$C_{45}H_{83}O_{13}P$	PI 36:2	0.5	1.10×10^{-3}	0.3	2.11×10^{-4}	1.3	2.37×10^{-04}	1.0	2.87×10^{-04}
885.5508	$C_{47}H_{83}O_{13}P$	PI 38:4	0.3	8.80×10^{-3}	0.2	1.54×10^{-3}	1.1	1.89×10^{-03}	0.9	2.04×10^{-03}
**M1-polymer**										
673.4821	$C_{37}H_{71}O_8P$	PA 34:1	0.7	8.89×10^{-05}	0.3	2.82×10^{-05}	0.7	5.38×10^{-05}	0.6	5.68×10^{-05}
747.498	$C_{43}H_{73}O_8P$	PA 40:6	0.9	9.41×10^{-05}	0.9	3.33×10^{-05}	1.0	5.92×10^{-05}	1.1	6.99×10^{-05}

751.5291	$C_{43}H_{77}O_8P$	PA 40:4	0.7	1.23×10^{-04}	0.6	4.20×10^{-05}	0.6	6.79×10^{-05}	0.8	7.18×10^{-05}
786.5294	$C_{42}H_{78}NO_{10}P$	PS 36:2	0.7	3.88×10^{-04}	0.6	1.68×10^{-04}	0.9	2.59×10^{-04}	0.8	2.88×10^{-04}
788.5451	$C_{42}H_{80}NO_{10}P$	PS 36:1	0.7	4.45×10^{-04}	0.8	1.87×10^{-04}	1.0	2.91×10^{-04}	0.9	3.26×10^{-04}
838.5609	$C_{46}H_{82}NO_{10}P$	PS 40:4	1.0	1.55×10^{-04}	1.0	6.06×10^{-05}	1.1	9.17×10^{-05}	1.1	1.05×10^{-04}
764.5244	$C_{43}H_{76}NO_8P$	PE 38:5	0.6	1.64×10^{-04}	0.7	7.38×10^{-05}	0.9	1.26×10^{-04}	0.8	1.47×10^{-04}
766.5401	$C_{43}H_{78}NO_8P$	PE 38:4	0.6	4.80×10^{-04}	0.6	2.41×10^{-04}	0.9	3.54×10^{-04}	0.8	4.04×10^{-04}
790.5402	$C_{45}H_{78}NO_8P$	PE 40:6	0.5	1.64×10^{-04}	0.7	7.06×10^{-05}	0.7	1.07×10^{-04}	0.7	1.22×10^{-04}
857.5196	$C_{45}H_{79}O_{13}P$	PI 36:4	0.8	5.39×10^{-04}	0.7	2.49×10^{-04}	1.0	3.66×10^{-04}	1.1	4.02×10^{-04}
861.5511	$C_{45}H_{83}O_{13}P$	PI 36:2	0.8	7.32×10^{-04}	0.9	3.43×10^{-04}	1.0	5.06×10^{-04}	1.0	5.52×10^{-04}
885.5508	$C_{47}H_{83}O_{13}P$	PI 38:4	0.5	4.77×10^{-03}	0.6	2.11×10^{-03}	0.7	3.18×10^{-03}	0.7	3.42×10^{-03}
**M2-polymer**										
673.4821	$C_{37}H_{71}O_8P$	PA 34:1	0.2	5.44×10^{-05}	0.2	8.07×10^{-05}	0	8.54×10^{-05}	0.3	9.54×10^{-05}
747.498	$C_{43}H_{73}O_8P$	PA 40:6	0.4	9.36×10^{-05}	0.6	1.54×10^{-04}	0.3	1.70×10^{-04}	0.6	2.70×10^{-04}
751.5291	$C_{43}H_{77}O_8P$	PA 40:4	0.1	1.04×10^{-04}	0.2	1.72×10^{-04}	0	1.77×10^{-04}	0.2	2.22×10^{-04}
786.5294	$C_{42}H_{78}NO_{10}P$	PS 36:2	0.1	1.36×10^{-04}	0.3	2.39×10^{-04}	0.2	2.32×10^{-04}	0.3	3.36×10^{-04}
788.5451	$C_{42}H_{80}NO_{10}P$	PS 36:1	0.3	7.07×10^{-05}	0.5	1.26×10^{-04}	0.3	1.11×10^{-04}	0.4	1.65×10^{-04}
838.5609	$C_{46}H_{82}NO_{10}P$	PS 40:4	0.3	1.42×10^{-04}	0.4	2.05×10^{-04}	0.4	2.08×10^{-04}	0.5	2.61×10^{-04}
764.5244	$C_{43}H_{76}NO_8P$	PE 38:5	0.2	2.29×10^{-05}	0.1	4.06×10^{-05}	0	4.36×10^{-05}	0.2	7.48×10^{-05}

766.5401	$C_{43}H_{78}NO_8P$	PE 38:4	0.3	2.31×10^{-04}	0.3	3.72×10^{-04}	0.1	3.65×10^{-04}	0.3	5.03×10^{-04}
790.5402	$C_{45}H_{78}NO_8P$	PE 40:6	0.3	9.42×10^{-05}	0.3	1.62×10^{-04}	0.1	1.65×10^{-04}	0.3	2.10×10^{-04}
857.5196	$C_{45}H_{79}O_{13}P$	PI 36:4	0.3	1.33×10^{-04}	0.3	2.31×10^{-04}	0.1	2.52×10^{-04}	0.4	4.50×10^{-04}
861.5511	$C_{45}H_{83}O_{13}P$	PI 36:2	0.4	1.80×10^{-04}	0.4	3.33×10^{-04}	0.2	3.45×10^{-04}	0.5	4.93×10^{-04}
885.5508	$C_{47}H_{83}O_{13}P$	PI 38:4	0.4	1.76×10^{-03}	0.3	2.97×10^{-03}	0.1	3.04×10^{-03}	0.3	4.78×10^{-03}

Table S4. Characteristic molecular ion and fragments of amino acid in 3D OrbiSIMS spectra (positive polarity)

Mass m/z	Assignment	Amino acids
80.0498	$C_5H_6N^+$	Leucine
86.0967	$C_5H_{12}N^+$	Isoleucine
81.045	$C_4H_5N_2^+$	Histidine
82.0528	$C_4H_6N_2^+$	Histidine
93.0449	$C_5H_5N_2^+$	Histidine
94.0527	$C_5H_6N_2^+$	Histidine

95.0605	$C_5H_7N_2^+$	Histidine
110.0713	$C_5H_8N_3^+$	Histidine
156.0768	$C_6H_{10}N_3O_2^+$	Histidine
100.087	$C_4H_{10}N_3^+$	Arginine
112.0869	$C_5H_{10}N_3^+$	Arginine
114.1026	$C_5H_{12}N_3^+$	Arginine
120.0444	$C_7H_6NO^+$	Tryptophan
130.0652	$C_9H_8N^+$	Tryptophan
131.073	$C_9H_9N^+$	Tryptophan
132.0808	$C_9H_{10}N^+$	Tryptophan
143.073	$C_{10}H_9N^+$	Tryptophan
157.0761	$C_{10}H_9N_2^+$	Tryptophan
158.0839	$C_{10}H_{10}N_2^+$	Tryptophan
159.0917	$C_{10}H_{11}N_2^+$	Tryptophan
84.0447	$C_4H_6NO^+$	Glutamic acid
84.0811	$C_5H_{10}N^+$	Lysine
86.0603	$C_4H_8NO^+$	Hydroxyproline
87.0555	$C_3H_7N_2O^+$	Asparagine

120.0808	$C_8H_{10}N^+$	Phenylalanine
166.0863	$C_9H_{12}NO_2^+$	Phenylalanine
101.071	$C_4H_9N_2O^+$	Glutamine
130.0499	$C_5H_8NO_3^+$	Glutamine
136.0758	$C_8H_{10}NO^+$	Tyrosine
104.053	$C_4H_{10}NS^+$	Methionine
116.0706	$C_5H_{10}NO_2^+$	Proline
82.0654	$C_5H_8N^+$	Multiple amino acids
83.0607	$C_4H_7N_2^+$	Multiple amino acids
88.0396	$C_3H_6NO_2^+$	Multiple amino acids
96.0809	$C_6H_{10}N^+$	Multiple amino acids
98.0966	$C_6H_{12}N^+$	Multiple amino acids
100.0394	$C_4H_6NO_2^+$	Multiple amino acids
102.055	$C_4H_8NO_2^+$	Multiple amino acids
107.0492	$C_7H_7O^+$	Multiple amino acids
114.055	$C_5H_8NO_2^+$	Multiple amino acids
117.0573	$C_8H_7N^+$	Multiple amino acids
118.0651	$C_8H_8N^+$	Multiple amino acids

119.0492	$C_8H_7O^+$	Multiple amino acids
128.0706	$C_6H_{10}NO_2^+$	Multiple amino acids
121.0648	$C_8H_9O^+$	Multiple amino acids
77.0389	$C_6H_5^+$	Generic fragment
80.0624	$C_6H_8^+$	Generic fragment
89.0388	$C_7H_5^+$	Generic fragment
91.0545	$C_7H_7^+$	Generic fragment
102.0465	$C_8H_6^+$	Generic fragment
103.0543	$C_8H_7^+$	Generic fragment
105.0699	$C_8H_9^+$	Generic fragment

Table S5. Peak exported from SurfaceLab positive mode from each tissue sample, consisting of ions detected in the spectrum and assigned as RG sequences of lysozyme $[M\text{-}H]^+$ $C_8H_{16}N_5O_2^+$, *m/z* 124.1298**.**

Sample	Area1	Area 2	Area 3	Area4

	Mass error p.p.m	Norm. intensity	Mass error p.p.m	Norm. intensity	Mass error p.p.m	Norm. intensity	Mass error p.p.m	Norm. intensity
**PDMS**	0.4	2.51×10^{-05}	0.6	4.09×10^{-06}	-0.1	2.30×10^{-06}	0.2	2.67×10^{-05}
**M1-polymer**	0.2	1.42×10^{-06}	0.3	5.59×10^{-05}	8.7	0	-6	0
**M2-polymer**	0.2	1.03×10^{-04}	-0.3	8.89×10^{-05}	-0.1	1.03×10^{-04}	0.2	1.43×10^{-04}

Table S6. Other small molecules in each sample and search by human metabolome data base.

Mass *m/z*	Assignment [M-H]$^-$	Metabolites	Area1		Area 2		Area 3		Area4	
			Mass error p.p.m	Norm. intensity	Mass error p.p.m	Norm. intensity	Mass error p.p.m	Norm. intensity	Mass error p.p.m	Norm. intensity
**PDMS**										
80.0497	$C_5H_6N^+$	Pyridine	4.1	9.77×10^{-05}	4.1	1.46×10^{-05}	3.9	2.67×10^{-05}	3.7	9.44×10^{-05}
81.0449	$C_4H_5N_2^+$	Pyrimidine	3.9	1.20×10^{-04}	4.2	2.19×10^{-05}	3.9	5.30×10^{-05}	3.6	1.22×10^{-05}
112.0869	$C_5H_{10}N_3^+$	Histamine	-0.1	5.95×10^{-04}	-0.2	1.90×10^{-04}	-0.2	1.96×10^{-04}	-0.4	5.76×10^{-04}

121.051	$C_5H_5N_4^+$	Purine	0.2	4.49×10^{-05}	0.4	5.31×10^{-06}	0.0	2.26×10^{-05}	-0.1	6.79×10^{-05}
**M1-polymer**										
80.0497	$C_5H_6N^+$	Pyridine	4.5	5.89×10^{-05}	4.5	2.57×10^{-05}	4.0	5.71×10^{-05}	3.5	1.08×10^{-05}
81.0449	$C_4H_5N_2^+$	Pyrimidine	4.0	5.87×10^{-05}	3.7	6.67×10^{-05}	4.1	5.03×10^{-05}	3.9	1.27×10^{-05}
112.0869	$C_5H_{10}N_3^+$	Histamine	0.0	1.70×10^{-04}	-0.1	7.32×10^{-05}	4.2	6.78×10^{-05}	4.0	3.03×10^{-05}
121.051	$C_5H_5N_4^+$	Purine	-0.1	2.13×10^{-06}	0.2	3.34×10^{-06}	-	0	-	0
**M2-polymer**										
80.0497	$C_5H_6N^+$	Pyridine	3.4	1.75×10^{-04}	3.2	9.20×10^{-05}	0	1.80×10^{-04}	0.3	2.61×10^{-04}
81.0449	$C_4H_5N_2^+$	Pyrimidine	3.1	2.39×10^{-05}	3.2	1.35×10^{-04}	3.3	3.36×10^{-04}	3.3	3.67×10^{-04}
112.0869	$C_5H_{10}N_3^+$	Histamine	-0.9	1.54×10^{-03}	-0.9	1.14×10^{-03}	-0.7	1.79×10^{-03}	-0.8	2.07×10^{-03}
121.051	$C_5H_5N_4^+$	Purine	-0.5	6.25×10^{-05}	-0.6	7.19×10^{-05}	-0.4	1.67×10^{-04}	-0.4	1.09×10^{-04}

Table S7. Chemical structure of the monomers and synthesis of copolymers, CHMA-co-DMAEMA and CHMA-co-iDMA.

Code	Monomer 1 name/structure (66%)	Monomer 2 name/structure (33%)	Copolymers

M1-polymer	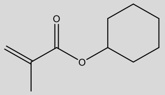<br>Cyclohexyl methacrylate<br>(CHMA)	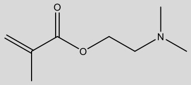<br>Dimethylamino-<br>ethylmethacrylate<br><br>(DMAEMA)	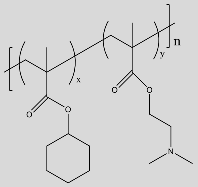<br>CHMA-co-DMAEMA
M2-polymer	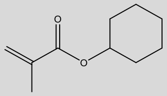<br>Cyclohexyl methacrylate<br>(CHMA)	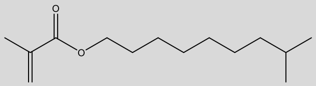<br>Isodecyl methacrylate<br><br>(iDMA)	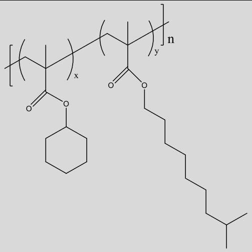

			CHMA-co-iDMA

Table S8. Hematoxylin and Eosin (H&E) staining schedule.

1.	Water rinse to remove OCT	5 min
2.	Haematoxylin solution	5 min
3.	Water rinse	1 min
4.	1 % acetic acid in alcohol	30 s
5.	Water rinse	1 min
6.	Alkine Scott’s	1 min
7.	Water rinse	1 min
8.	Eosin	2 min
9.	Water rinse	1 min
10.	50 % alcohol	30 s
11.	70 % alcohol	30 s

12.	90 % alcohol	30 s
13.	100 % alcohol × 2	30 s each
14.	Xylene × 2	2 min each
15.	Mounting media onto tissue slide and covered with a thin coverslip	

Table S9. Masson Trichrome Stain Kit (Light Green) Masson 1929 schedule**.**

1.	Fixing, 4 %PFA	1 h
2.	Water rinse	5 min
3.	Haematoxylin, mixing equal volumes of Weigerts solution A & B (1:1) as required	20 min
4.	Water rinse	1 min
5.	1 % acetic acid in alcohol	30 s
6.	Water rinse	1 min
7.	Ponceau fuchsin Masson solution for	5 min

8.	Rinse in distilled water	2 min
9.	The light green solution	3 min
10.	Water rinse	30 s
11.	50 % alcohol	30 s
12.	70 % alcohol	30 s
13.	90 % alcohol	30 s
14.	100 % alcohol × 2	30 s each
15	Xylene × 2	2 min each
16.	Mounting media onto tissue slide and covered with a thin coverslip	

Table S10. Sequential antibody staining for macrophage marker schedule**.**

1.	Washing in 0.2% Tween 20 in PBS × 3	5 min
2.	0.1% Triton X-100 in PBS	10 min

3.	Washing in 0.2% Tween 20 in PBS × 3	5 min
4.	5% BSA and plus 5% donkey serum in PBS	1 h
5.	0.2% PBS-Tween 20 rinse × 3	5 min
6.	Add diluted primary antibody with 1:50 of rabbit anti-mouse iNOS (Abcam) and 1:50 of goat anti-mouse Arg-1 (Thermo Fisher Scientific) in 5% goat serum at 4ºC	Overnight
7.	Washing in 0.2% Tween 20 in PBS × 3	5 min
8.	Add diluted secondary antibodies, donkey anti-goat IgG (H + L), and donkey anti-rabbit IgG (H + L) labelled with Alexa Fluor-594 and -488 (1:200; A11058 and A21206, Thermo Fisher Scientific),	1 h
9.	Washing in 0.2% Tween 20 in PBS × 3	5 min
10.	4',6 Diamidino-2-Phenylindole (DAPI, 20000 ng/ml)	5 min
11.	Washing in 0.2% Tween 20 in PBS × 2	5 min
12.	Final, washing in distilled water	5 min
13.	Mounting media onto tissue slide and covered with a thin coverslip	
